# Melanocytes—A Novel Tool to Study Mitochondrial Dysfunction in Duchenne Muscular Dystrophy

**DOI:** 10.1002/jcp.24290

**Published:** 2012-11-20

**Authors:** Camilla Pellegrini, Alessandra Zulian, Francesca Gualandi, Elisa Manzati, Luciano Merlini, Maria E Michelini, Luisa Benassi, Sandra Marmiroli, Alessandra Ferlini, Patrizia Sabatelli, Paolo Bernardi, Nadir M Maraldi

**Affiliations:** 1 Department of Medical Science, University of FerraraFerrara, Italy; 2 Department of Biomedical Sciences, University of PadovaPadova, Italy; 3 Laboratory of Musculoskeletal Cell Biology, IOR-IRCCSBologna, Italy; 4 Unit of Pediatric Surgery, University of FerraraFerrara, Italy; 5 Department of Dermatology, University of Modena and Reggio EmiliaModena, Italy; 6 Department of Histology, University of Modena and Reggio EmiliaModena, Italy; 7 CNR, National Research Council of Italy, IGM—IORBologna, Italy

## Abstract

Dystrophin is a subsarcolemmal protein that, by linking the actin cytoskeleton to the extracellular matrix via dystroglycans, is critical for the integrity of muscle fibers. Here, we report that epidermal melanocytes, obtained from conventional skin biopsy, express dystrophin with a restricted localization to the plasma membrane facing the dermal–epidermal junction. In addition the full-length muscle isoform mDp427 was clearly detectable in melanocyte cultures as assessed by immunohistochemistry, RNA, and Western blot analysis. Melanocytes of Duchenne muscular dystrophy (DMD) patients did not express dystrophin, and the ultrastructural analysis revealed typical mitochondrial alterations similar to those occurring in myoblasts from the same patients. Mitochondria of melanocytes from DMD patients readily accumulated tetramethylrhodamine methyl ester, indicating that they are energized irrespective of the presence of dystrophin but, at variance from mitochondria of control donors, depolarized upon the addition of oligomycin, suggesting that they are affected by a latent dysfunction unmasked by inhibition of the ATP synthase. Pure melanocyte cultures can be readily obtained by conventional skin biopsies and may be a feasible and reliable tool alternative to muscle biopsy for functional studies in dystrophinopathies. The mitochondrial dysfunction occurring in DMD melanocytes could represent a promising cellular biomarker for monitoring dystrophinopathies also in response to pharmacological treatments. J. Cell. Physiol. 228: 1323–1331, 2013. © 2012 Wiley Periodicals, Inc.

Duchenne muscular dystrophy (DMD) is caused by mutations in *DMD*, which encodes the protein dystrophin. Dystrophin is a subsarcolemmal protein that links the actin cytoskeleton to the extracellular matrix via the dystroglycan complex (DGC). It interacts with integral membrane proteins (sarcoglycan, dystroglycans, syntrophin, and dystrobrevin) that are assembled in the DGC (Muntoni et al., 2003). This complex forms a bridge across the sarcolemma and connects the basal lamina of the extracellular matrix to the inner cytoskeleton by binding F-actin *via* laminin 2. Muscle cells lacking dystrophin exhibit multiple defects, including abnormal fragility of the sarcolemma, elevated cytosolic Ca^2+^ levels, and increased oxidative stress (Petrof, 2002). In response to increases in intracellular Ca^2+^ concentrations, mitochondria can undergo a so-called “permeability transition,” due to an increased susceptibility of mitochondria to opening of the permeability transition pore (PTP) following stress (Zoratti and Szabo, 1995; Rasola and Bernardi, 2011). Mitochondrial dysfunction, due to PTP premature opening, has been found in some myopathies, including Ullrich congenital muscular dystrophy (UCMD; Irwin et al., 2003; Angelin et al., 2007) and limb-girdle muscular dystrophy (LGMD; Baghdiguian et al., 1999), and in *mdx* mice (Reutenauer et al., 2008). Importantly for potential therapeutic applications, mitochondrial depolarization showed a positive response to cyclophilin inhibitors, such as CsA (Angelin et al., 2008; Merlini et al., 2008) and Debio 025 (Reutenauer et al., 2008; Tiepolo et al., 2009). In an open pilot trial of five patients with collagen VI myopathies, orally administered CsA, ameliorated both the structural organization and the performances of dystrophic muscle fibers (Merlini et al., 2008).

Melanocytes are the pigment-producing cells of the epidermis. Each melanocyte at the basal layer of the epidermis is functionally connected to fibroblasts in the underlying dermis and to keratinocytes in the overlying epidermis. In human skin, melanocytes are localized at the dermal–epidermal junction (DEJ) in a characteristic regularly dispersed pattern (Yamaguchi et al., 2007). Melanocytes attachment to the DEJ is crucial for their role and involves laminin-binding receptors as integrins (Pinon and Wehrle-Haller, 2011) and dystroglycans (Herzog et al., 2004). Melanocytes express muscular 427 kDa full-length mRNA, and the mDp427 dystrophin is highly represented in untransformed primary melanocyte cultures (Korner et al., 2007). Therefore, we explored melanocytes as a potential surrogate model for muscle cells. We found that the mDp427 dystrophin isoform was expressed in melanocytes in vivo with a restricted localization to the plasma membrane facing the DEJ; DMD melanocytes displayed morphological alterations of mitochondria similar to those detected in dystrophin-deficient muscle cells and a latent dysfunction unmasked by inhibition of the ATP synthase. These data indicate that melanocytes represent a promising cellular model for monitoring the mitochondrial dysfunction in dystrophinopathies.

## Materials and Methods

### Patients

Skin and muscle biopsies from three healthy subjects and five DMD patients were collected; samples were frozen in isopentane pre-chilled and stored in liquid nitrogen. All patients were previously diagnosed by genetic, histochemical, and biochemical analysis. Patient DMD1 carried deletion of exon 51, patients DMD2 and DMD3 deletion of exon 45, patient DMD4 deletion of exons 45-52, and patient DMD5 a stop mutation in *DMD* gene. All participants provided written informed consent, and approval was obtained from the Ethics Committee of the University of Ferrara.

### Epidermal samples

Skin fragments from four healthy donors and two DMD patients were cut into small pieces and washed several times with DMEM and 1% antibiotics. The epidermis was mechanically separated from the dermis after overnight incubation in 0.5% dispase II (Roche Indianapolis, IN) at 4°C, and harvested with PBS (Kormos et al., 2011).

### Melanocyte and muscle cell cultures

Primary cultures of normal melanocytes were obtained from the leg skin of four healthy donors and two DMD patients. Cells were extracted from the epidermal samples by digestion with 0.25% tripsin–EDTA (Kormos et al., 2011), and maintained in M254 culture medium (GIBCO Life Technologies Ltd, Paisley, UK) supplemented with phorbol-12-myristate 13-acetate, transferrin, hydrocortisone, insulin, bovine pituitary extract, basic fibroblast growth factor, and fetal calf serum (HMGS supplement; GIBCO). Muscle cell cultures from tibialis anterior of one healthy subject and from DMD patients were established as previously reported (Angelin et al., 2007).

### Immunofluorescence analysis

Unfixed frozen sections (7-µm thick) of skin biopsies from healthy donors and DMD patients were incubated with rabbit polyclonal antibody which recognizes an internal region of dystrophin (amino acids 801-1100; Santa Cruz Biotechnology, Inc., Santa Cruz, CA,), detected with anti-rabbit TRITC-conjugated IgG (DAKO Denmark A/S, Glostrup, Denmark); mouse monoclonal DYS1, DYS2, DYS3, utrophin, β-dystroglycan (Novocastra Leica Biosystems GmbH, Nussloch, Germany) and α-dystroglycan (Upstate Technologies, UBI Merck Millipore Billerica, MA,) antibodies were revealed with secondary anti-mouse TRITC or FITC-conjugated antibodies (DAKO). Samples, when indicated, were double labeled with anti-pMEL-17 (Monosan, Uden, The Netherlands), cytokeratin (Sigma-Aldrich, St. Louis, MO), or laminin γ1 antibodies (Chemicon Merck Millipore Billerica, MA). Unfixed frozen sections of muscle biopsies from one healthy donor and DMD patients were incubated with DYS1, DYS2, and DYS3 antibodies as previously reported (Sabatelli et al., 2003). Samples were mounted with an anti-fading reagent (Molecular Probes Life Technologies Ltd, Paisley, UK) and observed with a Nikon epifluorescence microscope. Normal cultured melanocytes were fixed with methanol at −20°C for 7 min, washed with phosphate-buffered saline (PBS) and incubated with anti-dystrophin (Santa Cruz), anti-β-dystroglycan, anti-α-dystroglycan, and Ki-67 (Santa Cruz) antibodies, revealed with secondary anti-rabbit or anti-mouse TRITC or FITC-conjugated antibodies (DAKO).

### Western blot analysis

Normal cultured melanocytes and epidermal samples were washed three times with PBS and harvested with 100 µl of RIPA lysis buffer. Total proteins were quantified with a Bio-Rad DC Protein Assay Kit. One hundred micrograms of protein from each samples were boiled in loading buffer and separated onto a 6% polyacrylamide gel. The fractionated proteins were electroblotted onto a nitrocellulose membrane at 35 V overnight at 4°C; after blocking with 5% dry milk solution for 60 min at room temperature, the membranes were incubated with DYS1 (Arechavala-Gomeza et al., 2007), pMEL-17 (Monosan) and GAPDH (Merck Millipore Billerica, MA) primary antibody and with a secondary anti-mouse horseradish peroxidise (HRP)-conjugated antibody (diluted 1:10,000; Santacruz). Chemiluminescent detection of proteins was carried out with ECL detection reagent Kit (GE Healthcare UK Ltd, Amersham, Buckinghamshire, England) according to the supplier's instructions.

### Electron microscopy

Skin fragments were fixed with 2.5% glutaraldehyde in 0.1 M cacodylate buffer for 2 h, post-fixed with 1% osmium tetroxide and embedded in Epon 812 epoxy resin. Melanocytes and muscle cell cultures were grown onto uncoated well plates, fixed with 2.5% glutaraldehyde in 0.1 M cacodylate buffer for 2 h, post-fixed with 1% osmium tetroxide. After dehydration, cells were detached with propylene oxide, and embedded in Epon812 epoxy resin (Ognibene et al., 1999). Ultrathin sections were observed with a Philips EM400 electron microscope operated at 100 kV. The quantity of altered mitochondria was evaluated on three independent experiments, and expressed as mean percentage. At least 700 mitochondria for each sample were studied for statistical evaluation; data were analyzed according to Mann–Whitney's *U*-test, and the criterion for statistical analysis was *P* *<* 0.05.

### RT-PCR analysis of dystrophin transcripts

Total RNA was isolated from epidermal samples and melanocyte cultures using the Rneasy Kit (Qiagen Sciences, Germantown, MD) following the manufacturer's instructions. Before cDNA synthesis, RNA was treated with DNAse I (Roche). Reverse trascription (RT) was performed using random hexanucleotide primers and Superscript III enzyme (Invitrogen) according to the protocol supplied. All PCRs reactions were carried out in a volume of 25 µl containing the cDNA template and oligonucleotide primers designed to amplify the full-length and short dystrophin isoforms and utrophin A and B (sequence available upon request). PCR reactions were analyzed on agarose gels containing ethidium bromide prior to photography.

### Mitochondrial membrane potential assay

Mitochondrial membrane potential was measured based on the accumulation of tetramethylrhodamine methyl ester (TMRM, Molecular Probes; Angelin et al., 2007). Primary cultures of melanocytes and myoblasts obtained from a healthy donor and patients DMD2 and DMD3 were seeded onto 24-mm-diameter round glass coverslips and grown for 2 days in M254 culture medium with HMGS supplement (GIBCO). The medium was then replaced with serum-free M254 medium supplemented with 10 nM TMRM for 30 min, and cellular fluorescence images were acquired with an Olympus IX71/IX51 inverted microscope, equipped with a xenon light source (75 W) for epifluorescence illumination and with a 12-bit digital cooled CCD camera (Micromax, Princeton Instruments, New Jersey Trenton, NJ). Data were acquired and analyzed using Cell R Software (Olympus corporation, Tokio, Japan). For detection of fluorescence, 568 ± 25 nm band-pass excitation and 585-nm long-pass emission filter settings were used. Images were collected with exposure time of 100 msec using a 40×, 1.3 NA oil immersion objective (Nikon Instruments Europe BV, Amsterdam, The Netherlands). The extent of cell and hence mitochondrial loading with potentiometric probes is affected by the activity of the plasma membrane multidrug resistance pump. In order to normalize the loading conditions, in all experiments with TMRM the medium was supplemented with 1.6 µM CsH, which inhibits the multidrug resistance pump but not the PTP (Bernardi et al., 1999). At the end of each experiment, mitochondria were fully depolarized by the addition of 4 µM of the protonophore carbonyl cyanide-*p*-trifluoromethoxy-phenyl hydrazone (FCCP). Clusters of several mitochondria were identified as regions of interest, and fields not containing cells were taken as the background. Sequential digital images were acquired every 2 min and the average fluorescence intensity of all relevant regions was recorded and stored for subsequent analysis.

## Results

### Melanocytes express dystrophin at the interface with the dermal–epidermal junction

**Fig. 1:**
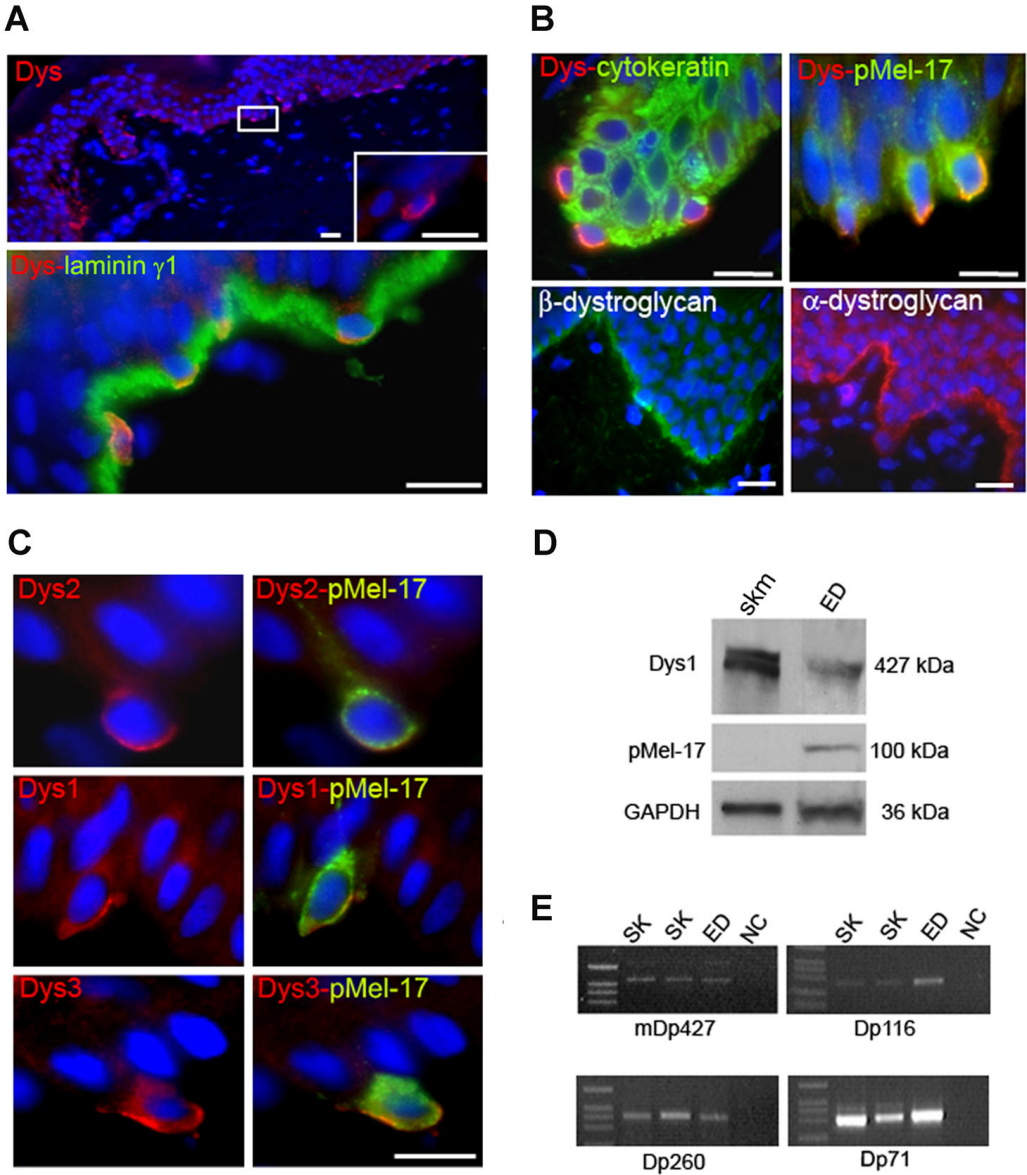
Dystrophin expression in the epidermis. A: Immunofluorescence analysis of dystrophin on perpendicular frozen sections of normal skin. Dystrophin is detected at the basal layer of the epidermis (upper part). The high magnification (inset) evidences the discontinuous labeling pattern at the DEJ. Double labeling with anti-laminin γ1 chain reveals that dystrophin localizes at interface with the DEJ, where it co-localizes with laminin γ1 chain (orange staining). Nuclear staining, DAPI. Bar, 50 µm. B: Immunohistochemical characterization of dystrophin-expressing cells. Double labeling with anti-dystrophin and specific markers of keratinocytes (cytokeratin) and melanocytes (p-MEL-17) showing that dystrophin labeling is restricted to melanocytes, while it is absent in keratinocytes. β-dystroglycan and α-dystroglycan localize at DEJ with a continuous pattern involving all the different cells at this side. Nuclear staining, DAPI. Bar, 50 µm. C: Immunofluorescence analysis of dystrophin with DYS2, DYS1, and DYS3 antibodies in perpendicular sections of normal skin. Melanocytes, identified by pMEL-17 antibody (green fluorescence) are double labeled with anti-dystrophin antibodies raised against carboxy-terminal (DYS2), internal (DYS1), and amino-terminal (DYS3) protein domain. Dystrophin staining is clearly detectable with all the antibodies, supporting the presence of the full-length isoform. Nuclear staining, DAPI. Bar, 20 µm. D: Western blot analysis of dystrophin in the epidermis (ED) and skeletal muscle (skm). DYS1 antibody reveals the presence of a band at 427 kDa in both samples; the amount of dystrophin in the epidermis is lower with respect to the normal skeletal muscle, possibly due to the relative low number of melanocytes present in the epidermis, as indicated by the low amount of melanocyte marker (pMEL-17). GAPDH has been used as loading control. E: RT-PCR analysis of dystrophin transcripts. RNA analysis of dystrophin isoforms in two skin samples (SK) and epidermis (ED) reveals the presence of muscle full-length mDp427 in all the samples examined. In addition, Dp260, Dp116, and Dp71 isoforms were also detected. NC, negative control.

Perpendicular sections of skin from normal subjects were immunolabeled with a polyclonal anti-dystrophin antibody, raised against the internal domain of the protein. In the epidermis, dystrophin labeling was detected in the basal layer, while it was absent in the stratum corneum and granulosum (Fig. 1A). Double labeling with anti-laminin γ1 chain, a marker of basement membrane, showed that dystrophin expression was restricted to the cell plasma membrane facing the DEJ. Interestingly, only a fraction of the cells of the basal layer expressed dystrophin (Fig. 1A). By double labeling with specific markers of keratinocytes (cytokeratin) and melanocytes (p-MEL17) dystrophin was selectively detected in melanocytes, while it was absent in basal keratinocytes (Fig. 1B). On the other hand, β-dystroglycan and α-dystroglycan, two dystrophin associated components, were homogeneously expressed along the epidermal basement membrane (Fig. 1B).

### Dystrophin isoforms in melanocytes

The immunohistochemical analysis of dystrophin with DYS1, DYS2, and DYS3 antibodies, specific for the dystrophin rod, C and N terminal domain, respectively, showed a strong labeling at the membrane of melanocytes (Fig. 1C). Western blot analysis with DYS1 antibody, which specifically recognizes the full-length isoform, was performed on a sample of normal epidermis: a band at 427 kDa was detected in the epidermis lysate, albeit at lower levels with respect to a normal skeletal muscle sample, possibly due to the relative low number of melanocytes present in the epidermis (Fig. 1D). RT-PCR analysis of dystrophin isoforms in skin epidermis confirmed the presence of muscle full-length mDp427. In addition, the Dp260, Dp116, and Dp71 shorter isoforms were also detected (Fig. 1E).

Dystrophin expression was also evaluated in primary melanocyte cultures from normal skin, both at the protein and transcript level. Previous studies described different patterns of dystrophin localization depending on the time of culture and cell proliferation in normal muscle cell cultures (Trimarchi et al., 2006). Thus, melanocytes were examined at 12, 48, and 72 h after plating. The rate of proliferation was determined by immunofluorescence analysis of Ki-67, a nuclear factor expressed exclusively in proliferating cells (Gerdes et al., 1983). At 12 h after plating melanocytes were all quiescent, in fact, they did not express Ki-67 (Supplementary Fig. 1); after 48 h, both quiescent and proliferating cells were present (not shown), while, at 72 h after plating all cells expressed Ki-67 indicating that they were actively cycling (Supplementary Fig. 1).

**Fig. 2:**
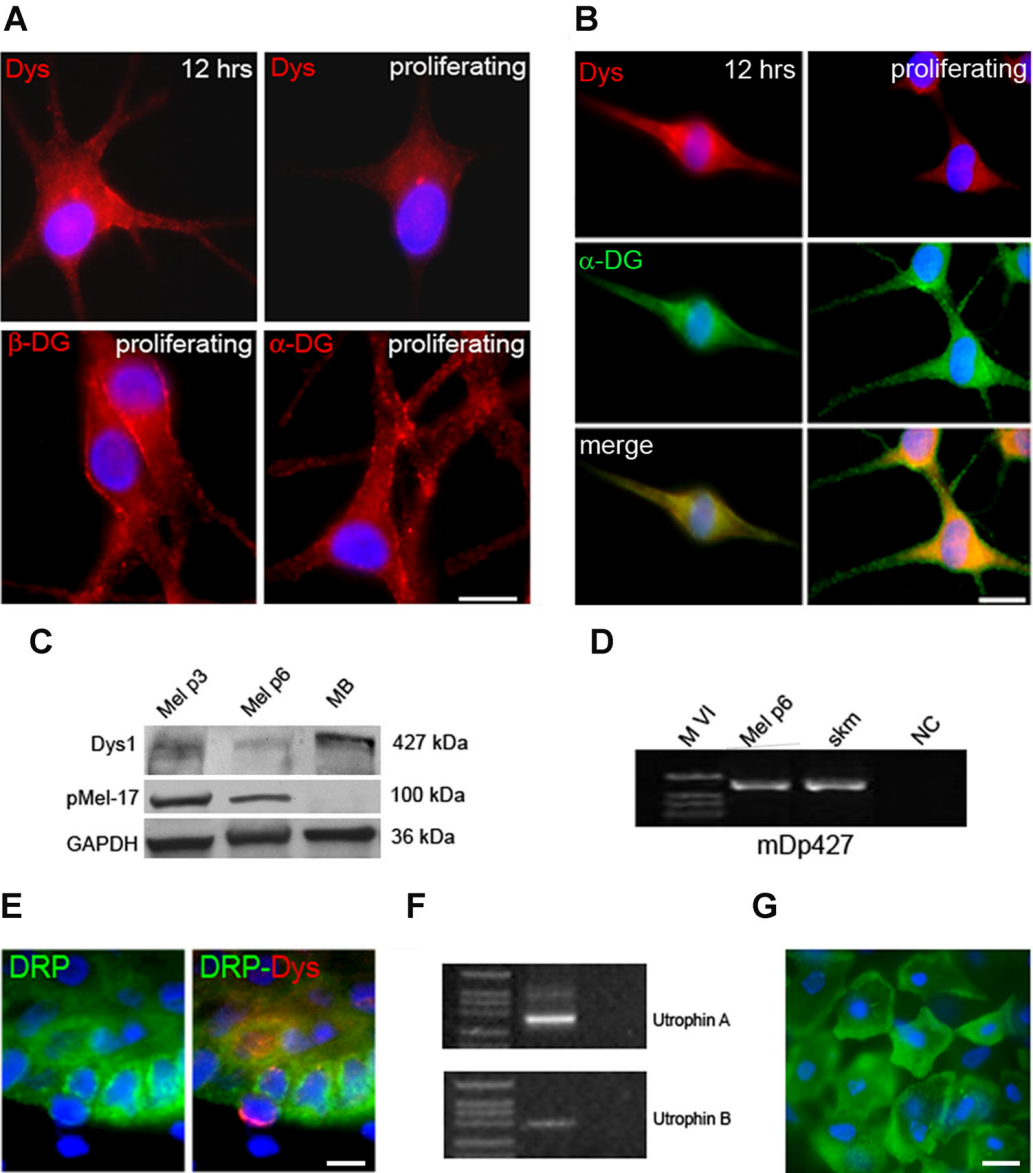
Dystrophin expression in cultured melanocytes. A: Melanocytes were grown onto coverslips for 12 or 72 h (proliferating). After 12 h dystrophin staining with DYS1 antibody (Dys) is detected at the membrane, while in proliferating cells, dystrophin localization is restricted to the perinuclear area. β-dystroglycan (β-DG) and α-dystroglycan (α-DG) show a membrane pattern in proliferating cells. Nuclear staining, DAPI. Bar scale, 50 µm. B: Double labeling with anti-dystrophin and anti-α-dystroglycan (α-DG) in melanocytes after 12 h of plating and in proliferating cells, clearly shows the difference of dystrophin pattern in different conditions of culture. Bar scale, 10 µm. C: Western blot analysis of dystrophin in cultured melanocytes at passage 3 (Mel p3) and 6 (Mel p6) and in differentiated myoblasts (MB) after 7 days in DMEM with 2% of FBS. DYS1 antibody detects a specific band in melanocyte samples at the same molecular weight of differentiated myoblasts. pMEL-17 was used as a marker of melanocytes, while GAPDH as loading control. D: RT-PCR analysis of mDp427 isoform in cultured melanocytes at passage 6 (Mel p6) and in skeletal muscle (skm) reveals the presence of muscle full-length mDp427 in both samples. NC, negative control. M VI, molecular weight marker VI. E: Immunohistochemical analysis of utrophin in perpendicular sections of normal skin. Utrophin (DRP, green fluorescence) is detectable in cells of the basal layer of the epidermis. Double labeling with anti-dystrophin antibody shows that dystrophin-positive cells do not express utrophin at the plasma membrane, indicating that dystrophin and utrophin are alternatively expressed in the basal epidermal cells. Nuclear staining, DAPI. Bar, 50 µm. F: RT-PCR analysis of utrophin in epidermis reveals the presence of the A and B isoforms. G: Primary keratinocytes obtained by epidermal layer digestion were seeded onto collagen I and examined for utrophin expression by immunohistochemistry. Utrophin is clearly detectable at the cell membrane with an homogeneous pattern. Nuclear staining, DAPI. Bar, 10 µm.

Dystrophin labeling pattern was different in quiescent cells, after 12 h of adhesion, with respect to proliferating; in fact, while in quiescent cells dystrophin was detected at the plasma membrane, in proliferating cells it translocated to the perinuclear area, with a cytoplasmic pattern (Fig. 2A). On the contrary, β-dystroglycan and α-dystroglycan membrane localization was maintained in proliferating melanocytes, both at the cell body and dendritic processes (Fig. 2A). Double-labeling with anti-dystrophin and anti-α-dystroglycan antibodies clearly showed the difference of dystrophin pattern in quiescent versus proliferating cells (Fig. 2B).

The expression level of dystrophin, evaluated by Western blot analysis with DYS1 antibody, indicated that the amount of the protein present in melanocytes at passage 3 is similar to that in differentiated myoblasts (Fig. 2C). The reduction observed at passage 6 could be associated with in vitro dedifferentiation (Kormos et al., 2011). RNA analysis in cultures showed a clearly detectable full-length muscle isoform (Fig. 2D).

### Utrophin, the homologue of dystrophin, is expressed by keratinocytes

In normal skin, utrophin was detectable in dystrophin-negative cells of the basal layer of the epidermis, while it was absent in dystrophin-expressing cells, suggesting that utrophin is alternatively expressed to dystrophin in basal keratinocytes (Fig. 2E). The expression of utrophin in the epidermis was also supported by RT-PCR analysis, which revealed the presence of the A and B isoforms (Fig. 2F). The evaluation of utrophin expression in primary keratinocytes cultures showed a clear membrane labeling with anti-utrophin antibody (Fig. 2G), while dystrophin was absent (not shown).

### Immunohistochemical and mophological characterization of dystrophin-deficient melanocytes in skin biopsies of DMD patients

**Fig. 3:**
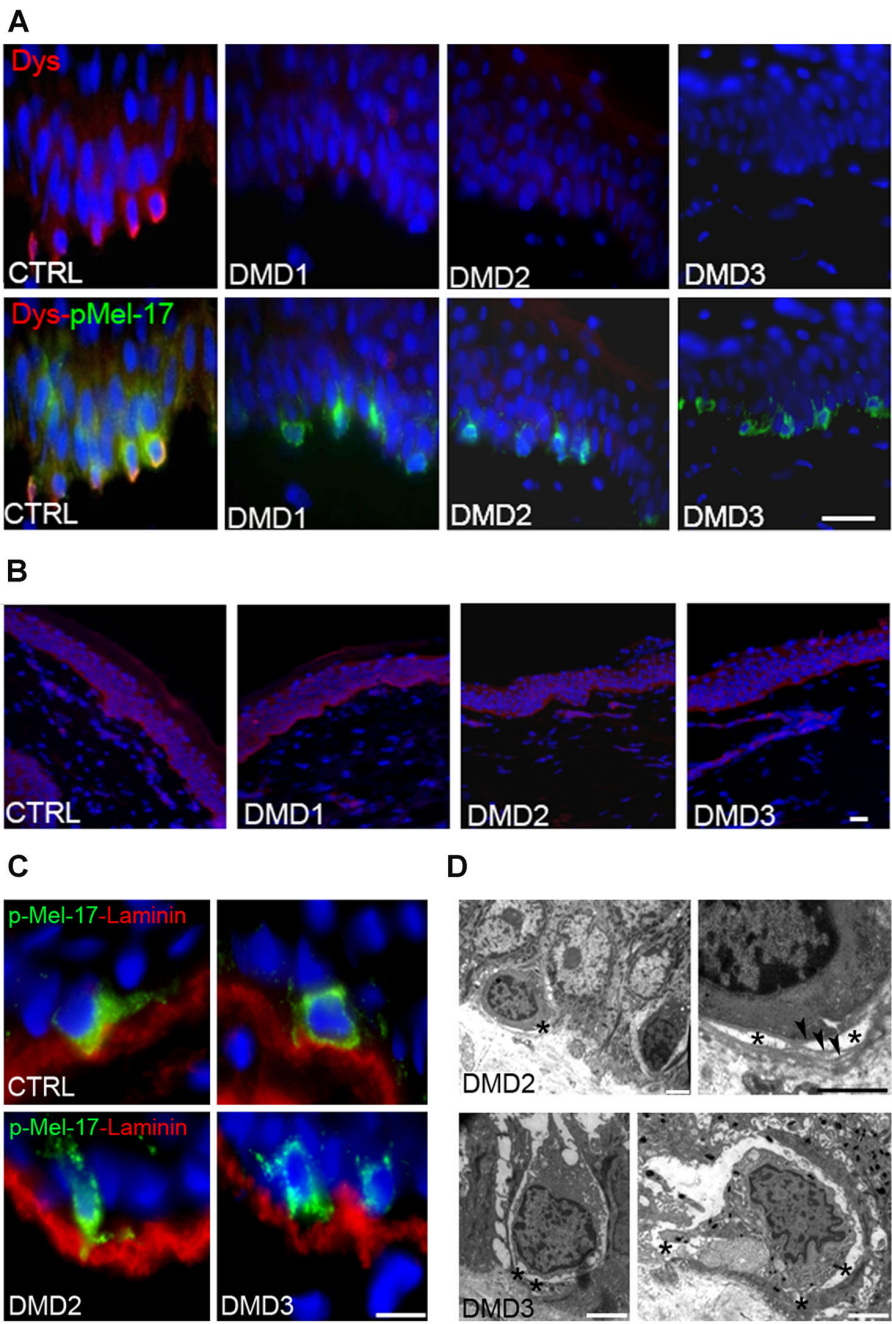
Immunofluorescence and ultrastructural studies of DMD skin biopsies. A: Dystrophin (Dys1 antibody) is not detectable at DEJ of DMD patients, despite the high number of epidermal melanocytes (p-MEL positive cells, green fluorescence). Nuclear staining, DAPI. Bar, 50 µm. B: Utrophin is normally expressed in the epidermis of DMD patients. Nuclear staining, DAPI. Bar scale, 50 µm. C: Double labeling with pMEL-17 and laminin γ1 in the skin biopsies of normal and patients DMD2 and DMD3. DMD melanocytes localize in the basal layer of the epidermis, similarly to normal melanocytes, however, they display an altered shape and a reduced surface of attachment to the DEJ (C). D: Ultrastructural analysis of skin sections from patients DMD2 and DMD3. Patients melanocytes appear detached from the basement membrane of the DEJ (asterisks); in some areas, the DEJ basement membrane appears duplicated (arrowheads). Bar, 3 µm.

Skin biopsies obtained from five genetically characterized DMD patients were analyzed for dystrophin expression. Dystrophin was absent at DEJ, despite the high number of epidermal melanocytes detectable at the basal layer (p-MEL positive cells; Fig. 3A, Supplementary Fig. 2A). Dystrophin was also absent in the arrector pili smooth muscle cells and myoepithelial cells of dermis (not shown). Dystrophin absence in DMD skin correlated with the expression pattern in muscle biopsies of the same patients, with the exception of rare (<1%) dystrophin-positive revertant fibers (Supplementary Fig. 2B), which could result from alternative skipping leading to the restoration of a functional protein (Klein et al., 1992).

Utrophin has been reported to be up-regulated in muscle cells of DMD patients with a possible compensative role (Karpati et al., 1993). The evaluation of utrophin in DMD skin samples, however, did not reveal differences in the staining pattern when compared with a normal control (Fig. 3B).

**Fig. 4:**
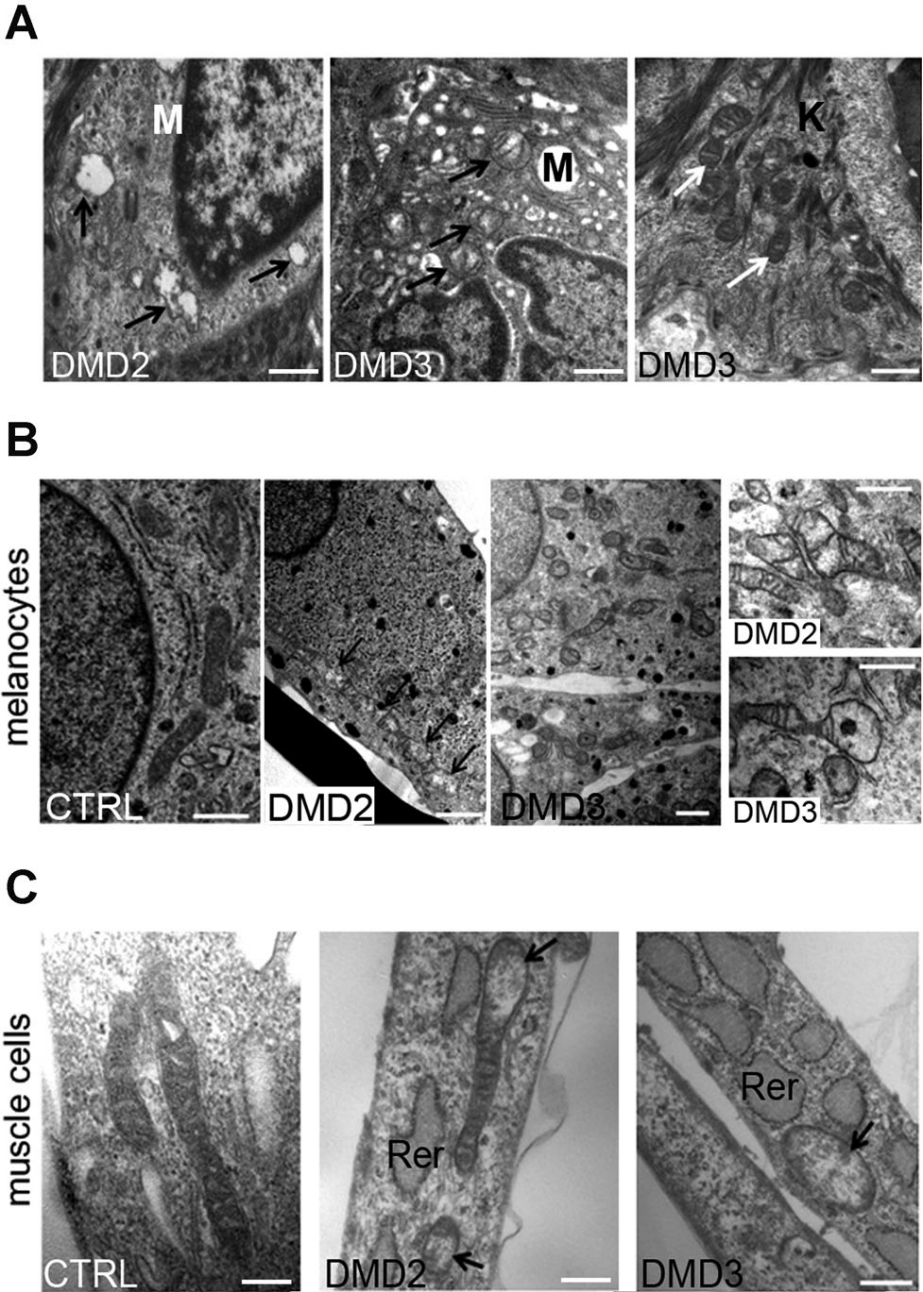
Morphological alterations of DMD mitochondria. A: Transmission electron microscopy analysis of DMD melanocytes (M) in skin sections show mitochondrial changes, such as increased size, reduced matrix density and disrupted cristae, (black arrows). On the contrary, mitochondria appears normal (white arrows) in DMD keratinocytes (K). B: Ultrastructural analysis of normal and DMD melanocytes. Normal melanocytes (CTRL) display elongated mitochondria with well-preserved cristae and dense matrix. Mitochondria of patients DMD2 and DMD3 appear enlarged with reduced matrix density and swelling (arrows). Bar, 600 nm. C: Ultrastructural analysis of normal and DMD myoblasts. Normal myoblasts (CTRL) show long mitochondria with a regular short axis. DMD2 and DMD3 cultured myoblasts show reduced matrix density and swelling (arrows). Rer, rough endoplasmic reticulum. Bar, 600 nm.

To evaluate whether dystrophin absence may affect melanocyte attachment to the DEJ, we performed double labeling with pMEL-17 and laminin γ1 in skin biopsies of DMD patients. Dystrophin-deficient melanocytes localized in the basal layer of the epidermis, similarly to normal melanocytes, however, they displayed an altered shape and a reduced surface of attachment to the DEJ (Fig. 3C). The ultrastructural analysis revealed the presence of duplication of the basement membrane in areas of the DEJ underlying melanocytes; aspects of detachment of melanocyte plasmamembrane from the basement membrane were commonly found (Fig. 3D). By contrast, DMD keratinocytes appeared attached to the DEJ basement membrane (not shown). Mitochondrial changes, including increased size, reduced matrix density and disrupted cristae, consistent with swelling, were also frequently found. On the other hand, mitochondrial alterations were not detected in DMD keratinocytes (Fig. 4A) and in melanocytes of normal skin (not shown). The presence of mitochondrial alterations was confirmed in melanocyte cultures of the same DMD patients (Fig. 4B). DMD mitochondria appeared generally enlarged; in longitudinal sections, multiple focal swelling areas were detected along their major axis (Fig. 2E). The percentage of altered mitochondria was 32% in DMD2 (SD ± 8.5, *P* < 0.005) and 38% (SD ± 9.7, *P* < 0.005) in DMD3 patient's melanocytes. On the other hand, mitochondrial abnormalities were only occasionally detected in melanocytes of healthy subjects. We compared melanocytes with myoblasts cultures from the same DMD patients by ultrastructural analysis. Interestingly, muscle cell mitochondria displayed focal swelling, similarly to melanocytes (Fig. 4C). The percentage of swollen mitochondria was 3% (SD ± 0.2), 12% (SD ± 3.4), and 15.7% (SD ± 2.1) in normal, DMD2, and DMD3 muscle cells, respectively.

### Latent mitochondrial dysfunction in melanocytes and myoblasts from DMD patients

**Fig. 5:**
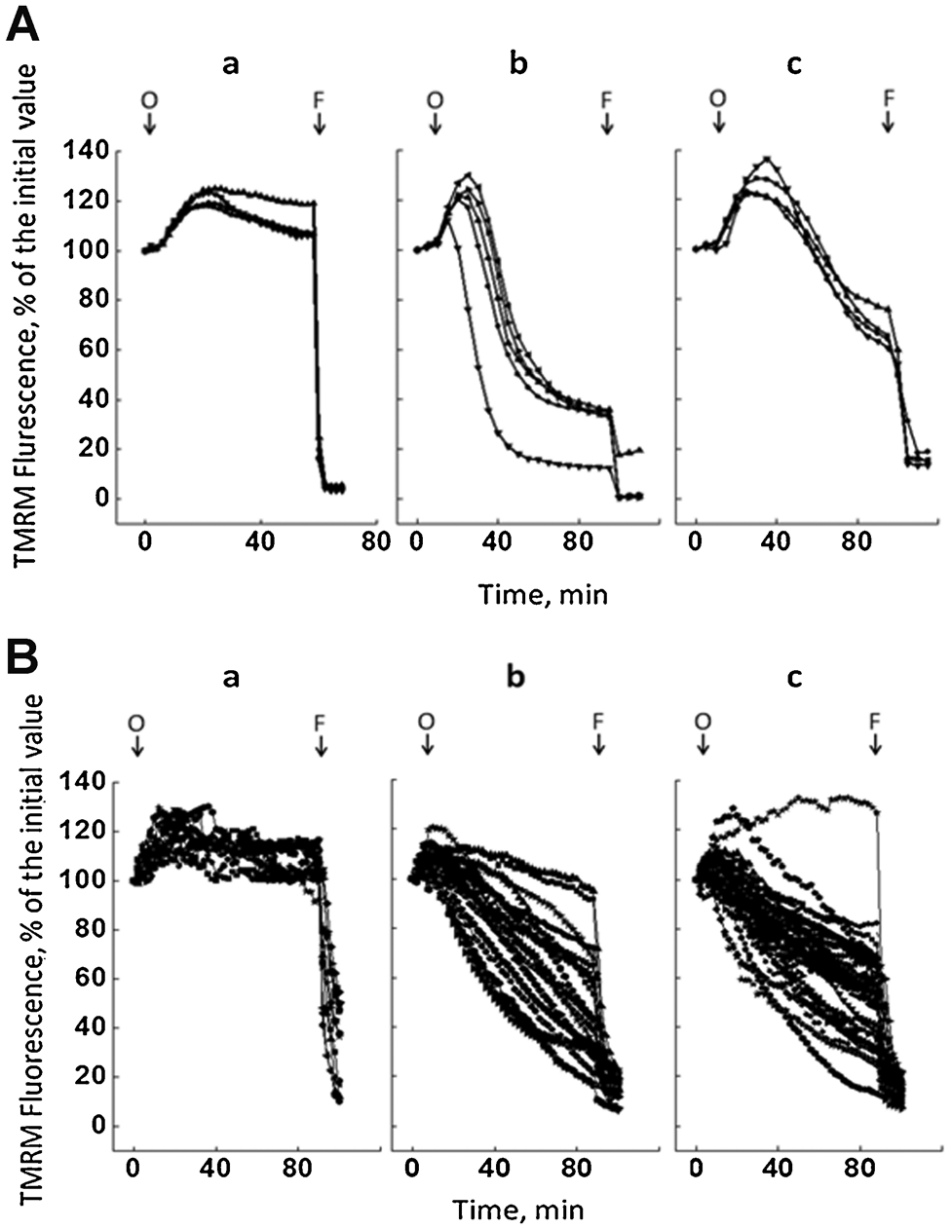
Functional alterations of mitochondria in DMD cultured melanocytes and myoblasts. A: Analysis of the effect of oligomycin on mitochondrial membrane potential in melanocytes from healthy donor (part a) and from patients DMD2 (part b) and DMD3 (part c). When indicated by arrows 4 µM oligomycin (O) and 4 µM FCCP (F) were added. Traces report one representative experiment of eight (part a), five (part b) and four (part c). Each line corresponds to one individual cell. Note that all cells from DMD patients but not cells from the normal donor depolarized with oligomycin. B: Analysis of the effect of oligomycin on mitochondrial membrane potential in myoblasts from healthy donor (part a) and from patients DMD2 (part b) and DMD3 (part c). When indicated by arrows 4 µM oligomycin (O) and 4 µM FCCP (F) were added. Each trace reports the response of one individual cell from 4 (part a), 9 (part b), and 12 (part c) experiments. If a threshold is set at 90% of the initial fluorescence, the fraction of myoblasts with depolarizing mitochondria is 0/12 (0%) for the normal donor, 18/20 (90%) for patient DMD2, and 33/35 (95%) for patient DMD3.

To test whether a functional defect of mitochondria could underlie the ultrastructural alterations, we studied mitochondrial function in primary cultures of melanocytes and myoblasts from one normal donor and patients DMD2 and DMD3. Mitochondria readily accumulated TMRM, indicating that they are energized irrespective of the presence of dystrophin. On the other hand, and at variance from mitochondria of normal donor (Fig. 5A,B—a part), upon the addition of the F_0_F_1_ ATPase inhibitor oligomycin mitochondria of DMD patients readily depolarized after the expected hyperpolarization (Fig. 5A,B—b and c parts), suggesting a latent dysfunction (e.g., Ca^2+^ deregulation; Angelin et al., 2008) that can be unmasked by inhibition of the ATP synthase.

## Discussion

Our investigation revealed several new findings. First, melanocytes, but not adjacent keratinocytes, express the dystrophin full-length isoform both in vivo and in vitro, and the protein is absent in melanocytes from DMD patients. Second, DMD melanocytes display morphological alterations of mitochondria similar to those detected in dystrophin-deficient muscle cells and a latent mitochondrial dysfunction unmasked by inhibition of the ATP synthase.

Interestingly, we found that melanocytes express dystrophin with a restricted localization to the plasma membrane facing the DEJ. Epidermal melanocytes are polarized cells, with basal and apical different functions which are recapitulated by differential composition of the membrane at different sides (Pinon and Wehrle-Haller, 2011). DEJ consists of a sheet-like structure, composed by extracellular matrix proteins, which forms an adhesion interface between epidermal basal cells (keratinocytes and melanocytes) and the underlying extracellular matrix. The DEJ acts as a permeability barrier; it controls cell organization and differentiation by mutual interactions between cell-surface receptors and molecules in the extracellular matrix (Santiago-Walker et al., 2009). DEJ exhibits features common to muscle cell sarcolemma. In particular, laminin alpha2 chain (Sewry et al., 1996), and dystroglycans (DGs) alpha and beta subunits have been detected at the DEJ (Herzog et al., 2004). In epithelia, DGs are essential for basement membrane formation (Barresi and Campbell, 2006) and are involved in maintenance of epithelial cell polarity (Durbeej et al., 1995; Michele et al., 2002; Masuda-Hirata et al., 2009), providing a link with the extracellular matrix through the alpha-dystroglycan glycosylated epitope. The specific localization we have found of dystrophin in melanocytes, and utrophin in basal keratinocytes, may indicate a specific role of these proteins at this side including the link between the dystroglycan and the actin cytoskeleton.

It has been recently reported that high levels of the Dp427m protein are expressed in primary human melanocytes, whilst very reduced amounts of the protein are detectable in melanoma cell lines (Korner et al., 2007). The muscle-specific full-length isoform (*Dp427m*) is highly expressed in skeletal and cardiac muscles, and at a reduced level, in Purkinje cerebellar neurons (Muntoni et al., 2003).There are several other tissue-specific isoforms of dystrophin, some exclusively or predominantly expressed in the brain or the retina (Waite et al., 2012). Here we show that, in addition to the Dp427m, melanocytes express the Dp116, Dp260, and Dp71 short isoforms. Dp260 has been reported in high concentrations in the retina, where it coexists with the full-length brain and muscle isoforms (D'Souza et al., 1995), while the Dp116 is only expressed in adult peripheral nerves (Byers et al., 1993); Dp71 is detected in most non-muscle tissues including brain, retina, kidney, liver, and lung and is present in cardiac but not skeletal muscle (Bar et al., 1990).

The dystrophin expressed by human melanocyte cultures, analyzed at both the mRNA and protein level, corresponds to the muscle-specific full-length mDp427 isoform, as previously reported (Korner et al., 2007). Interestingly, dystrophin continues to be expressed when melanocytes are maintained in culture; however, whilst the protein is retained at the plasma membrane in resting adherent melanocytes, its localization at the peri-nuclear cytoplasm characterizes proliferating melanocytes.

Whilst dystrophin is expressed by normal epidermal melanocytes, it is absent in the epidermis of DMD patients as well as in the muscle biopsies of the same patients. The absence of dystrophin, both in muscle and skin melanocytes, is in agreement with the type of mutations and with the frame-shift hypothesis (Koenig et al., 1989). However, it remains to be explained why reversion of dystrophin expression was not noted in DMD melanocytes. It has been reported that the spontaneous restoration of reading-frame of dystrophin occurring in some muscle fibers (revertant) correlates with degeneration/regeneration cycles (Yokota et al., 2006) indicating that this mechanism could be muscle specific, as a consequence of the dystrophic process.

When DMD skin biopsies are analyzed at the ultrastructural level, melanocytes appear detached with respect to the basement membrane of the DEJ. These data suggest that dystrophin may act in stabilizing melanocyte adhesion to the basement membrane and that this function is impaired in DMD patients. A previous study showed DMD gene deletions in melanoma cell lines; the occurrence of DMD mutations correlated with increased migration, whereas re-expression of DMD attenuated the phenotype (Korner et al., 2007). Our data support the hypothesis that dystrophin may critically change the adhesion and migratory capacity of melanocytes.

The second finding of the present investigation is related to the observation that DMD melanocytes show characteristic alterations of the mitochondrial morphology, consistent with swelling, which are not present in the neighboring keratinocytes. These mitochondrial alterations persist in melanocytes in culture conditions, arising the question of whether they are mechanistically linked to disease pathogenesis, as is the case for collagen VI-related muscular dystrophies that involve inappropriate PTP opening (Irwin et al., 2003).

We evaluated mitochondrial function in primary melanocyte and myoblast cultures. In cells from healthy donor the addition of the F_0_F_1_ ATPase inhibitor oligomycin caused hyperpolarization, as expected of phosphorylating cells. Indeed, in respiring cells the mitochondrial membrane potential is maintained by proton pumping through the respiratory chain, and ATP synthesis draws a fraction of the proton gradient, which therefore increases when the ATP synthase is blocked. Strikingly, the initial hyperpolarization induced by oligomycin was instead followed by fast depolarization in melanocytes and myoblasts from the two DMD patients. This behavior is reminiscent of the anomalous response we detected in myoblasts from UCMD patients (Angelin et al., 2007) and suggests a latent mitochondrial dysfunction in DMD cells like the one found in mdx mouse model (Pauly et al., 2012). Indeed, the initial hyperpolarization indicates that mitochondria are respiring and making ATP normally, yet oligomycin initiates a set of events ending in fast depolarization. For the first time here we show that a similar mechanism may be responsible for mitochondrial alterations in both DMD melanocytes and myoblasts, which would be worsened by the increased oxidative stress found in dystrophin-deficient cells (Menazza et al., 2010).

The idea that Ca^2+^-dependent mitochondrial dysfunction is a causative event in onset of DMD has been put forward as early as in 1976 (Wrogemann and Pena, 1976), and recently reinforced by the partial rescue from muscle pathology observed after inhibition of mitochondrial cyclophilin D, a positive effector of the PTP, with Debio025 in *mdx* mice (Millay et al., 2008; Wissing et al., 2010).

Skin biopsy as a diagnostic tool in dystrophinopathies has been already reported, as dystrophin is expressed at the plasma membrane of arrector pili smooth muscle cells (Tanveer et al., 2009; Ferlini et al., 2010). However, the use of this method remains uncommon since the uneven distribution of arrector pili and the localization of myoepithelial cells in deep dermis requires large and deep biopsies. Our finding of dystrophin expression by epidermal melanocytes make skin biopsy an appealing source for dystrophin detection, and may represent an extremely useful tool to monitor the effect of therapeutic treatments. In addition, considering that melanocyte cultures can be easily obtained by conventional skin biopsy and that pure melanocyte cultures can be obtained by using selective culture medium (Kormos et al., 2011), they may represent a feasible and reliable tool alternative to muscle biopsy for functional studies like mitochondrial dysfunction in dystrophinopathies.
