# Supplementary figures and images for: Melanocytes—A Novel Tool to Study Mitochondrial Dysfunction in Duchenne Muscular Dystrophy
Source: J Cell Physiol. 2012 Nov 20;228(6):1323–31. doi: 10.1002/jcp.24290 (PMC3601437; doi:10.1002/jcp.24290)

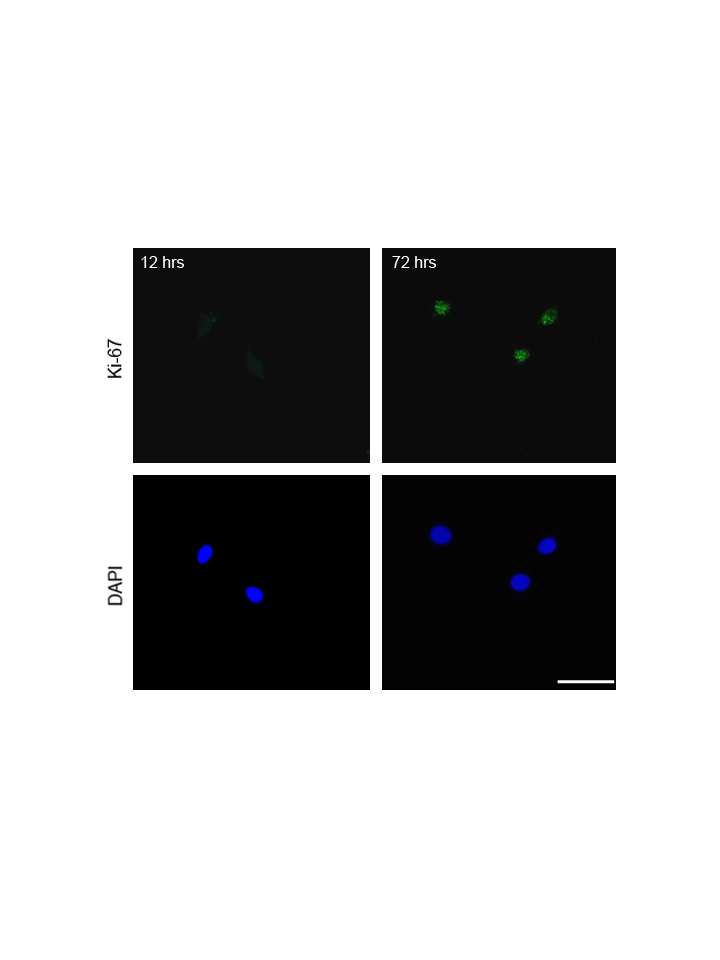

Supplement: Supplementary file 1 [file jcp0228-1323-SD1.tif]

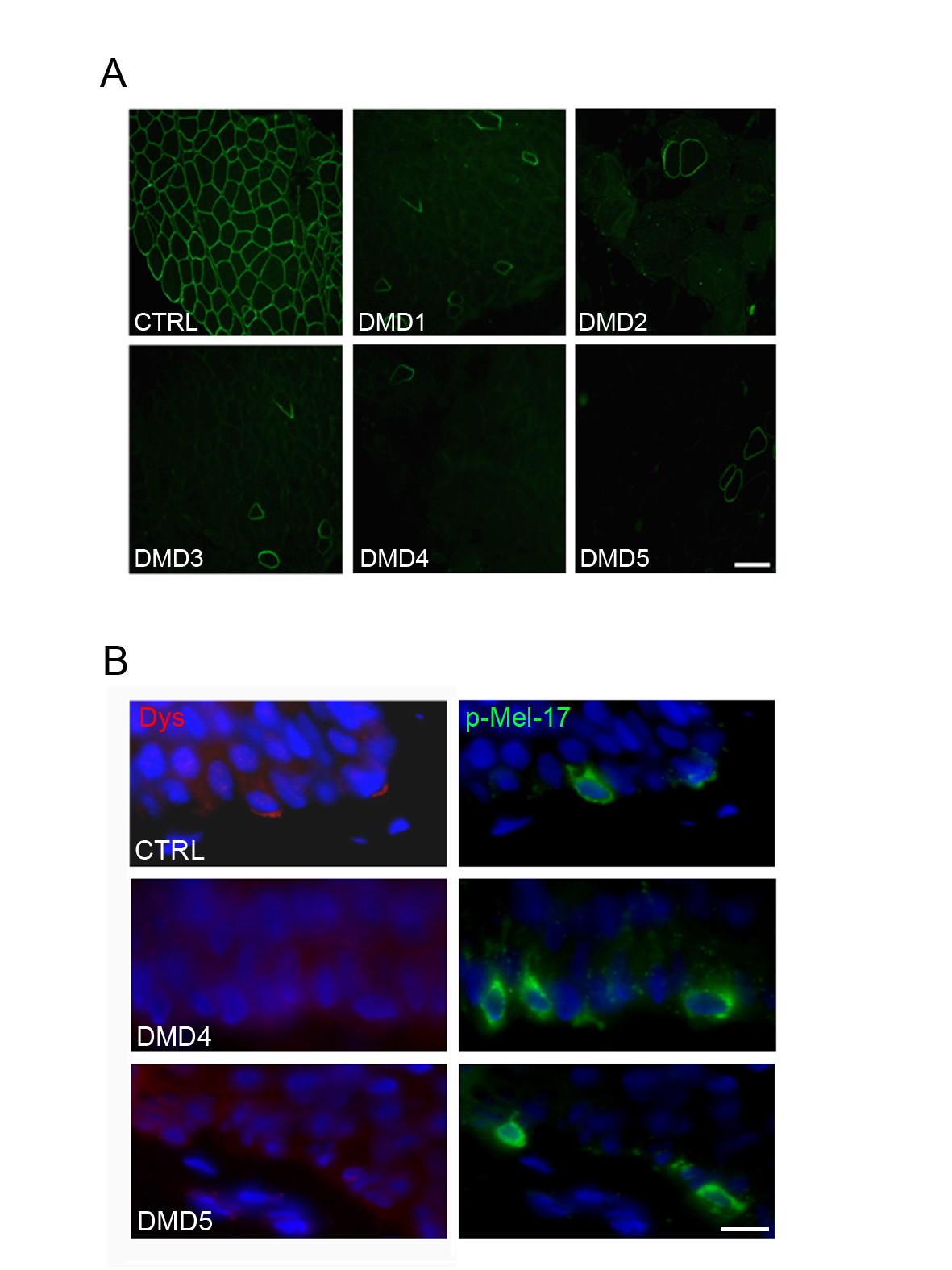

Supplement: Supplementary file 2 [file jcp0228-1323-SD2.tif]
