# Supplementary material for: Melanocytes—A Novel Tool to Study Mitochondrial Dysfunction in Duchenne Muscular Dystrophy
Source: J Cell Physiol. 2012 Nov 20;228(6):1323–31. doi: 10.1002/jcp.24290 (PMC3601437; doi:10.1002/jcp.24290)
Supplement: Supplementary file 3 [file jcp0228-1323-SD3.doc]

**Supplementary Fig. 1.** Immunofluorescence analysis of Ki-67 in normal melanocyte cultures at 12 and 72 hours after plating. After 12 hours, melanocytes did not express Ki-67 nuclear factor, suggesting that they are quiescent; at 72 hours all melanocytes appear proliferating, as indicated by the positivity for Ki-67 antibody . Bar, 20 μm.

**Supplementary Fig. 2. (**A**)** Immunofluorescence analysis of dystrophin in muscle biopsies of an healthy subject (CTRL) and patients DMD1, DMD2, DMD3, DMD4 and DMD5. Dystrophin was absent in most muscle fibers of DMD patients, with the exception of few “revertant fibers”. Bar scale, 50 μm. (B) Immunofluorescence analysis of dystrophin in skin biopsies of an healthy subject (CTRL) and patients DMD4 and DMD5, showing the absence of dystrophin labeling at the plasma membrane of patient melanocytes. Bar scale, 20 μm.
